# Prices for Ingenuity

**DOI:** 10.1371/journal.pbio.0030288

**Published:** 2005-08-16

**Authors:** Bill O'Neill

## Abstract

Competitions and community exchanges are spurring scientific progress.

Martin Reese proved that he was more than a fly on the wall in the Drosophila Genome Center at the University of California at Berkeley when, as a young PhD student, he encouraged colleagues to approach their work in an entirely new way. The goal was to develop efficient computational tools to predict individual genes from the masses of genetic code being sequenced. “The problem was people published papers on their algorithms…on different data sets,” recalls Reese, “so it was very hard to get a really good assessment on actually whose is the best and what are really the right methods and the right underlying signs to be used to do this job right.” Borrowing an idea from protein biologists, Reese brought together 12 teams under the umbrella of the Genome Annotation Assessment Project (GASP), provided them with the same Drosophila sequence to hone their prediction programs, and invited them to defend their results at a workshop in Heidelberg, Germany, a few months later, in July 1999. What the participants lacked in funding, they made up for, in abundance, with bonhomie and commitment, says Reese. And the timing could not have been better. The newly available Drosophila genome allowed Reese to test the workshop's top two programs on the front line: “We learned some tricks through this experiment [and] the Drosophila was one of the best annotated genomes as a result” (Figure 1).

**Figure 1:**
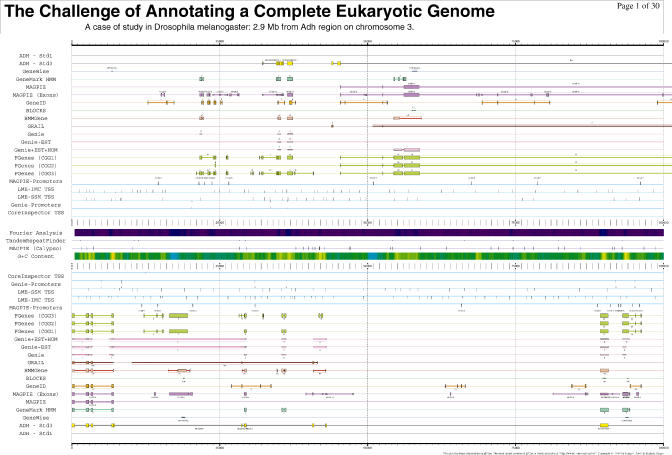
Drosophila melanogaster Genome Sequence from the 1999 GASP Project (http://www.fruitfly.org/GASP) The expert annotations of the Berkeley Drosophila Genome Project groups are on top and on the bottom of the display (yellow). The submitted predictions from 12 different groups are in different colours, and close to the centre, general DNA sequence features are shown. (Image: Reese et al. [1])

Exploiting competitive spirit to advance research is not new (Box 1), though it more often comes with the prospect of lucrative prizes from entrepreneurs eager to pit technical brains against each other to secure fast-track solutions. Such competitions are now more common and quirkily diverse, from battlefield strategists looking for a new generation of robotic navigators, to transport planners desperate for cooler travel underground, to NASA chiefs eager to extract oxygen from moon dust. Since 2003, there's been the Methuselah Mouse Prize (M Prize) for extending life span, and the X Prize Foundation, established in 1995, has just introduced an annual cup competition for space-travel innovations. The 55-year-old challenge to find a computer that can respond like a human still attracts competitors for an annual award, and even the world's climate-change negotiators proposed a prize for innovation ahead of the G8 Economic Summit in July, before seeing geopolitics squash the idea (along with a boost in research and development). The granddaddy of competitions—the prize launched in 1714 to determine longitude at sea—is these days heralded as the coming of the modern business world's entrepreneurial spirit to science.

**Box 1. Prize Competitions**

**X Prize Cup**

A prospective annual award for space-travel innovations from the X Prize Foundation, to replace its Ansari X Prize, whose $10 million purse for flying a private vehicle at least twice into space and back again within a fortnight went to US aviator Burt Rutan in October 2004. The award follows a long line of aviation prizes that go back to and beyond Charles Lindbergh's historic solo flight across the Atlantic in *Spirit of St. Louis* in 1927, which won him $25,000 from New York hotelier Raymond Orteig.

**M Prize**

An ongoing challenge that consists of two awards: a Longevity Prize for the oldest Mus musculus (currently standing at 1,819 days), and a Rejuvenation Prize for the best late-onset intervention (based on the rejuvenated mouse's age at death, currently standing at 1,356 days). Awards come from a fund, currently valued at around $1.3 million, to which anyone may contribute. A leading sponsor is “The 300”—modelled on the 300 Spartans who crucially delayed the invasion of Greece by hordes of Persians in 480 B.C.—whose members pledge regular contributions to the fund and whose names will be etched in history (as those of the Spartans were engraved on a stone tablet in Sparta).

**Loebner Prize for Turing Test**

British mathematician Alan Turing postulated, in 1950, that a “thinking” computer could produce responses to fool an interrogator that it was human; the prize, created by New Jersey industrialist Hugh Loebner in 1990, keeps the Turing Test a live challenge. Loebner has pledged $100,000 (plus a gold medal) for the first computer whose responses are indistinguishable from a human's. In the meantime, an annual prize of $3,000 (plus a bronze medal) goes to the most human computer that year. In 2005, according to Loebner, the award of $25,000 (plus a silver medal) looks likely to be won for the first time.

**Longitude Prize**

This prize was established in 1714 by the British government to determine longitude at sea. Instead of relying on astronomical sightings, watchmaker John Harrison built a precision clock to keep the time of a home port (of known longitude). Denied the £20,000 prize by assessors (wary that astronomy had been bypassed), Harrison petitioned King George III to circumvent them and to persuade Parliament to award him directly. Harrison was finally rewarded in 1773, 12 years late and 45 years after he began work on his “marine chronometer”. He died on his birthday in 1776, at the age of 83.

**Moon Regolith Oxygen Challenge**

Just this year, NASA announced a deadline stretching into 2008 for its third Centennial Challenge competition, the Moon Regolith Oxygen Challenge, to extract breathable oxygen from simulated lunar soil, and is dangling a purse of $250,000 in front of likely duellists.

Such prizes can bask in ambitious publicity campaigns and cash incentives, running into millions of dollars, in an effort to tap the broadest seam of ingenuity, yet money and marketing, even military muscle, are no guarantee of success. The Defense Advanced Research Projects Agency is having to rerun the Grand Challenge it staged late last year to find the fastest “autonomous ground vehicle” that can cover around 175 miles of treacherous desert track in under ten hours. Unperturbed, the Defense Advanced Research Projects Agency has doubled the prize money to $2 million. London Underground, which runs the capital's subterranean train service, has just abandoned its Cooling the Tube contest, after spending nearly two years appraising 3,500 entries from 60 countries and failing to find any feasible innovation to cope with summer heat waves that leave Tube travellers sweltering. And London Mayor Ken Livingstone, who promised £100,000 to the winner in 2003, has kept his hands in his pockets.

Against this background, the astonishing success of GASP and of a flourishing brand of similar low-key and often low-budget exchanges among scientific researchers, each vying to devise the perfect algorithm for some knotty problem, in fields that range from protein biochemistry to information retrieval to statistical genetics, seems all the more remarkable. And yet more remarkable still about these research exchanges is the avowed disdain among participants for the term “competition” to describe what they prefer to call a “community experiment”, and their claimed satisfaction (on coming first) with fame and glory among peers rather than with any cash award.

## Critical Assessments—“It's Not a Competition”

As someone who could be in the running for ageing research's M Prize, Jim Carey, a population biologist and Professor of Entomology at the University of California at Davis, explains the problem with many such contests: “It is not so much that legitimate ageing researchers do not want to be seen as actively seeking a prize, as it is that a research strategy built on the goal of winning the prize would be way too high a risk.” Carey has considered his chances though. “When I heard about [the M Prize] we were in the midst of our ovary transplant mouse studies and it crossed my mind momentarily that maybe we'd be in the running. But it had no influence on our thinking about the type of research we do; this is not what drives us. If we were to ever win this prize (hypothetically), it would be by default rather than by inspiration; that is, we would claim the prize (since why not?) but that would not have been the driving force.”

At the community-experiment level, however, goals can be much more clearly tied to practical research agendas (Box 2). Among the most successful participants is David Baker, Professor of Biochemistry at the University of Washington in Seattle. Baker is involved in exchanges to develop computational methods to predict three-dimensional protein structure, the Critical Assessment of Techniques for Protein Structure Prediction (CASP), and protein–protein interactions, the Critical Assessment of Predicted Interactions (CAPRI), in which the correct results have been obtained experimentally but are known only to assessors. The most accurate prediction is declared winner.

**Box 2. Community Experiments**

**CASP—Critical Assessment of Techniques for Protein Structure Prediction**

Research teams make blind predictions about the structures of the same set of proteins from given sequences of amino acids. Started in 1994 and staged every two years, the experiments are coordinated by Lawrence Livermore National Laboratory in Livermore, California. For CASP6 last year, more than 200 teams from 24 countries provided over 30,000 predictions on 90 protein domains. John Moult founded CASP in response to the “clear inadequacy of the peer reviewed publication system in this area of biology [and to] new ways of doing things made possible by cheap universal electronic communication”. An associated Web-based community discussion arena, FORCASP (Forum for CASP), provides an online meeting place and an intense discussion venue for the CASP community.

**CAPRI—Critical Assessment of Predicted Interactions**

Research teams make blind predictions about the structures of protein–protein complexes from given structures of the individual proteins. CAPRI aims to do for macromolecular interaction, a central theme in functional genomics, what CASP has done for protein structure. Started in 2001 and staged whenever an experimentalist offers an adequate target, according to cofounder Joël Janin, CAPRI is coordinated by the European Bioinformatics Institute at Hinxton, United Kingdom. Round seven began in May. Over four years, X-ray crystallographers have provided 21 targets, including two that were cancelled.

**CAFASP—Critical Assessment of Fully Automated Structure Prediction**

Evaluates the performances of automatic prediction servers to determine how accurately they predict protein structures without the intervention of experts (as allowed in CASP), such that nonexperts could use them with confidence.

**CAMDA—Critical Assessment of Microarray Data Analysis**

Research teams analyse the same standard datasets and compare notes on the different techniques to mine microarray data. Modelled on CASP, CAMDA was founded in 2000 at Duke University Bioinformatics Shared Resource in Durham, North Carolina, and has staged conferences every year since then.

**TREC—Text Retrieval Conference**

Founded in 1992 by the National Institute of Standards and Technology and the US Department of Defense, TREC is a series of workshops (TREC 2005 reports in November) to encourage research in information retrieval from large text collections, notably for the benefit of the intelligence community. A more recent initiative, supported by the US National Science Foundation, focuses on the study of the retrieval of genomic data, which is broadly interpreted to mean not just gene sequences but also supporting documentation such as research papers and laboratory reports.

BioCreAtIvE—Critical Assessment of Information Extraction Systems in Biology

Established in 2003 at the National Center of Biotechnology (Centro Nacional de Biotecnología) in Madrid, Spain, BioCreAtIvE claims to be the “first very biologically motivated evaluation of text mining systems” [2].

**GAWs—Genetic Analysis Workshops**

Started in 1982 and now under the auspices of the International Genetic Epidemiology Society, GAWs bring genetic epidemiologists together to evaluate statistical methods on real or computer-simulated data that organizers distribute to investigators about six or seven months before the next meeting. GAW15 is scheduled for November 2006.

**GASP—Genome Annotation Assessment Project**

Some 12 groups participated in a one-off experiment in 1999, coordinated by the Drosophila Genome Center at the University California at Berkeley to assess gene and functional site predictions in genomic DNA using a Drosophila sample. Earlier this year, the Municipal Institute of Medical Research (Institut Municipal d'Investigavió Mèdica) in Barcelona, Spain, launched E-GASP (in association with the Encyclopedia of DNA Elements project), which challenged 18 teams to do the same for the human genome.

Success with computational modelling promises much. Better algorithms to predict the locations of genes would make finding them much less time-consuming and would lead to the discovery of more of them, insists Reese. The problem for geneticists is the lack of a robust comparison against which to gauge the accuracy of their predictions. “The protein people have the three-dimensional structure, which is clear,” he says. For Baker, determining protein structures experimentally is expensive and time-consuming, and “cannot keep up with the explosion of DNA sequences,” he says. “If we could accurately and consistently predict protein structures and interactions, it would have a huge impact on biology.

**Figure 2:**
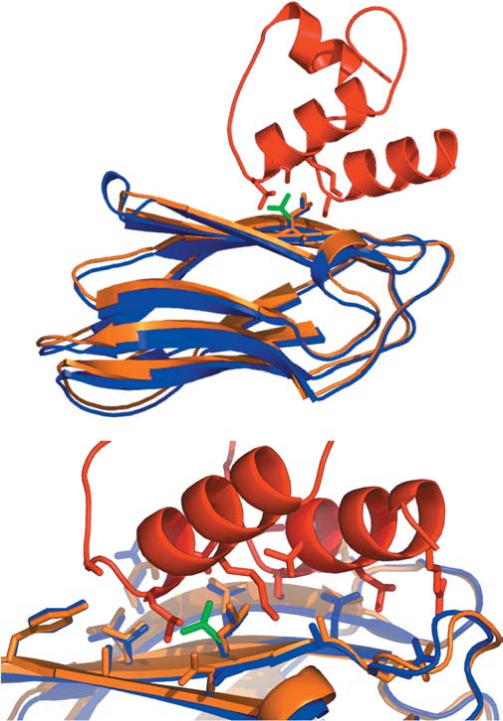
Structure Prediction with RosettaDock in CAPRI Prediction of the structure of the dockerin–cohesin complex (Target 12). Superposition of predicted (blue) and X-ray (red and orange) protein complex structures. A side chain whose conformation was correctly predicted to change upon complex formation is shown in green. The upper panel shows the whole complex; the lower panel shows details of the interface. In addition to the rigid-body orientation, the conformation of most of the side chains is predicted correctly, leading to the correct identification of 87% of the contacts in the crystal structure. (Illustration: Created by Ora Furman, University of Washington, using the PyMOL Molecular Graphics System [http://www.pymol.org])

“The most exciting results so far in any of these things for me personally were our results in the last CAPRI test,” says Baker. “The predictions were so stunningly accurate that if we'd made [them] inside my research group…I'd have been convinced that we must have cheated somehow,” he recalls. “Several predictions were much more accurate than any predictions of anything in structural biology have ever been” (Figure 2).

## Revolutionising Research (in Some Areas at Least)

Like Reese with respect to genetics, Baker sees the evolution of community experiments, in the form of CASP and CAPRI, as rescuing structural biologists from primary research papers that cannot be trusted. Too many published models rely on known results: “It's like trying to predict yesterday's weather from conditions that you knew the day before,” he says. “It's not conscious cheating, it's just that if you're trying to reproduce some set of [known] results with a computational model, if you try hard enough and you're smart enough then you'll figure out a way to do it.” The issue, he adds, is “whether you actually have captured some essential truth about how things work or whether you've just managed to twiddle all the numbers so that you reproduce a certain set of results.”

He's now convinced that the days of depending on experimentation alone to determine protein biochemistry are numbered, as the benefits of CASP and CAPRI kick in: “And a good measure of when we are there is these types of experiments.” In a manuscript in review, Baker reports that “for about a third of the small proteins we looked at—very small proteins, less than 85 amino acids—we could predict their structures quite accurately. You'd like it to be 100%…but it's a lot better than it was a few years ago when it'd have been zero.” For the moment, he says, lack of sufficient computational power is the problem.

John Moult, who founded CASP, agrees that reliable prediction is not far off. “I always say five years. Been saying that for about 20 years now,” he notes. “Seriously—if we can get effective refinement methods, then homology models based on high sequence identity (say, more than 30%) could quickly become competitive with experiment. However, we are only just beginning to progress on that problem, so it is hard to call.”

CASP is succeeding where similar collaborations to resolve other biological questions could easily struggle, notes Moult, Professor of Computational Biology at the Center for Advanced Research in Biotechnology at the University of Maryland Biotechnology Institute in Rockville. “The CASP model requires new experimental data to become available on an appropriate time scale [and] that's fairly uncommon,” he says. “On the other hand, it is my strong conviction that new communication methods will allow a whole range of new ways of collaborating on a community scale.”

## Granted, There Can Be Issues

But the collaboration is not always an easygoing affair. “Overall it works well, but there can of course be tensions in various forms,” says Moult. Participants who feel that the evaluation criteria are unfair to their predictions present the most common complaint: “Though this has happened, it is the exception,” he stresses. And growing sensitivity at funding agencies about the value of community experiments increases the tension. Involvement in CASP improves a researcher's chances of securing a grant, “or rather, not being involved in CASP may damage prospects,” says Moult. “This is not a good thing. It puts pressure on people to participate whether they think it's a good idea or not. I also suspect the significance of the results is sometimes overestimated by review committees.”

Such friction may be more of an issue in the US than in Europe, according to Joël Janin, Professor of Biophysics at the Centre National de la Recherche Scientifique Laboratory of Structural Enzymology and Biochemistry near Paris, France, and CAPRI's cofounder. “CAPRI wasn't planned to be a competition, and I do my best to keep the 'community-wide experiment' spirit in it,” he notes. “This seems to work in Europe and Asia for the moment, but American participants tell me they feel pressure from grant agencies.” The National Institutes of Health funds CASP, while CAPRI runs on a shoestring. “When Shoshana Wodak [from the Free University of Brussels] and I launched CAPRI, there was skepticism from our colleagues in the US that it could be run from Europe,” recalls Janin. “That skepticism was partly justified—we failed to get EU funding, and in the end Shoshana is moving from Brussels to Toronto.”

On the edge of these life sciences communities, looking in, is Ellen Voorhees, a computer scientist, who runs the Text Retrieval Conference (TREC) at the National Institute of Standards and Technology in Gaithersburg, Maryland, US. Since 2003, a decade after it was established, TREC has expanded its annual research workshops on improving the effectiveness of information retrieval systems, notably for the benefit of the intelligence community, to include assessments of methods for recovering genomics data.

Voorhees appreciates the tensions with community experiments. While TREC assessors do evaluate different retrieval systems and publish scores, she says, “TREC offers no award, and names no winners.” But some people still call TREC a competition, and there is an undeniable competitive element to it, she admits. “I used to try to correct people who called TREC a competition, but have given that up as a hopeless task.”

## Benchmarking Research

Voorhees appreciates the rewards of community experiments. “TREC has created a series of retrieval test collections that define benchmark tasks that drive the research. These collections simply could not have been built without a collaborative effort because the collections depend on the pooled results of many different retrieval systems. A single organisation trying to build a collection of similar size could not obtain a collection of equivalent quality because of the bias introduced by a single system.

“Retrieval effectiveness doubled on the basic ‘ad hoc’ task over the first six years of TREC,” notes Voorhees. “TREC introduced the first large-scale evaluations of cross-language retrieval, and retrieval of recordings of speech. More than 250 groups from more than 20 countries have participated in at least one TREC. Many groups have participated multiple times. These groups must see some value in participating.”

With community experiments proving to be such a valuable tool across computational research, could they also help to solve questions other than algorithmic ones? Baker is far from convinced: “Prediction experiments are special for prediction problems, which will generally be computational.” Janin agrees: “I cannot imagine how to organize a wet bench experiment in the same way, but who knows?” Moult is more accommodating: “Things like CASP have so far focussed on testing how well computational methods succeed at reproducing experimental reality. In that mode, [it is] hard to see how experiment might fit in,” he says, adding wryly, “experiment reproducing experiment?” But the next generation of community experiments could see more of an overlap with experiment, he concedes: “For example, asking the small molecule docking community to contribute suggestions as to what might be the ligand binding specificity of proteins of unknown function. These suggestions would then help guide experimental binding studies. This sort of community-wide computational experiment does have more similarity with some community-based experimental projects, for example, Tom Terwilliger's TB structure consortium [http://www.doe-mbi.ucla.edu/TB/], where he manages target and results lists, and in principle, anyone can do the needed experimental work.” But for Reese, who now runs Omicia, a prognostic genetics start-up he founded in 2002 in Emeryville, California, community experiments have a finite life. “Once we have all the genes found, then [GASP] will become redundant.”

## Abbreviations

CAPRI: Critical Assessment of Predicted Interactions
CASP: Critical Assessment of Techniques for Protein Structure Prediction
GASP: Genome Annotation Assessment Project
M Prize: Methuselah Mouse Prize
TREC: Text Retrieval Conference
